# LncRNA-mediated early regulatory networks in sugar beet (*Beta vulgaris* L.) response to low nitrogen

**DOI:** 10.1186/s12864-025-12301-6

**Published:** 2025-11-14

**Authors:** Qing Bai, Kehua Chen, Haodi Ji, Yuanhang Zhou, Xiaodong Li, Dali Liu, Wang Xing

**Affiliations:** 1 National Beet Medium-Term Gene Bank, Heilongjiang University, Harbin, 150080 China; 2 Key Laboratory of Beet Genetics and Breeding, College of Modern Agriculture and Ecological Environment, Heilongjiang University, Harbin, 150080 China; 3 Engineering Research Center of Agricultural Microbiology Technology, Ministry of Education & Heilongjiang Provincial Key Laboratory of Plant Genetic Engineering and Biological Fermentation Engineering for Cold Region & Key Laboratory of Molecular Biology, College of Heilongjiang Province & School of Life Sciences, Heilongjiang University, Harbin, 150080 China; 4 Manas Agricultural Experimental Station, Xinjiang Academy of Agricultural Sciences, Changji, 832200 China; 5 Inner Mongolia Academy of Agricultural & Animal Husbandry Sciences, Huhhot, 010031 China; 6 China National Seed Group Co., Ltd., Sanya, 572000 China

**Keywords:** Sugar beet (*Beta vulgaris* L.), Long non-coding RNAs (lncRNAs), Low nitrogen response, Competing endogenous RNA (ceRNA) networks, Plant hormone signal transduction

## Abstract

**Background:**

Sugar beet (*Beta vulgari*s L.) is a globally important sucrose-producing crop. As a “nitrogen (N)-responsive species”, it specifically relies on precise N management to maximize agroeconomic potential. However, excessive N application reduces sucrose accumulation efficiency and elevates non-sucrose constituents despite increasing root biomass. Clarifying the molecular regulatory mechanisms underlying low nitrogen (LN) response is therefore essential for improving nitrogen use efficiency (NUE) of sugar beet.

**Results:**

Comparative transcriptomics of sugar beet germplasm ‘780016B/12 Superior’ under normal (CV, 5 mmol/L N) and low nitrogen (LN, 0.5 mmol/L N) conditions after 12 h of treatment identified 120 and 254 differentially expressed long non-coding RNAs (DELs) in foliage and roots, respectively. Functional annotation of 1,454 long noncoding RNA-message RNA (lncRNA-mRNA) pairs (trans/cis = 3.47:1) revealed the coordinated regulation of DEL-target genes in nitrogen metabolism, transmembrane transport, and plant hormone signal transduction. Within these LN responsive networks, lncRNAs of XR_791134.2 and LNC_011801 functioned as key components, which correlated with glutamine synthetase (*GS2*)-mediated ammonium assimilation and auxin transporter-like protein (AUX2/3/4) suppression redirecting nitrogen resources, respectively. Additionally, competing endogenous RNA (ceRNA) networks further integrated hormonal signaling with nitrogen sensing. Specifically, lncRNA LNC_016830 is situated at a critical junction point, that interacted with miR396a/b-5p to regulate auxin-sensitive transcription factors *GRF7/9* and coordinated ABA signaling through *CRWN3*-mediated *ABI5* degradation. Crucially, most of ceRNA-associated mRNAs were targets of growth-suppressing hormones, including the brassinosteroid receptor SR160, a dual regulator that links lncRNA networks to steroid-mediated stress responses.

**Conclusions:**

This study reveals lncRNAs as key correlates balancing nitrogen assimilation and developmental plasticity in sugar beet, and provides molecular targets for breeding high NUE cultivars.

**Supplementary Information:**

The online version contains supplementary material available at 10.1186/s12864-025-12301-6.

## Introduction

Sugar beet (*Beta vulgaris* L.), a globally significant sucrose-producing crop, accounts for approximately 25% of annual worldwide sugar production [1]. As a ‘nitrogen (N)-responsive species’, this crop demonstrates strict dependence on precise N management to maximize its agroeconomic potential. Research indicates that while increased N application enhances root biomass production, it simultaneously reduces beet sucrose accumulation efficiency and elevates concentrations of non-sucrose constituents in root tissues [2]. This nutritional paradox indicates the urgent need to develop innovative strategies for enhancing nitrogen use efficiency (NUE) and to systematically elucidate the molecular mechanisms underlying low-nitrogen tolerance and high-efficiency nitrogen utilization in sugar beet.

Nitrogen, a fundamental macronutrient alongside carbon and oxygen, serves as a building block for proteins and nucleic acids, driving cellular growth and metabolic homeostasis [3]. Plants primarily assimilate inorganic N as nitrate (NO₃⁻) and ammonium (NH₄⁺), which dominate bioavailable soil N pools and regulate developmental processes [4, 5]. Nitrate transporters (NRTs) and ammonium transporters (AMTs) cooperatively regulate nitrogen uptake, so that these ions are transported via nitrite/ammonium (NRT/AMT) transporter networks and assimilated through the glutamine synthetase (GS)/glutamate synthase (GOGAT) pathway, initiating downstream and regulatory cascades. N deficiency disrupts this cycle, suppressing key enzyme activity [6], and impairing photosynthesis through Calvin cycle inhibition [7]. To adapt to such low N (LN) environment, plants establish positive transcriptional reprogramming, for example: *Zm*EREB98 modulates nitrate uptake efficiency through NRT1.1 regulation in *Zea mays* [8], while *Arabidopsis thaliana At*Dof1.7 mediates LN stress responses by activating *At*NRT2.4 expression [9]. Many evidence further implicates non-coding RNAs (ncRNAs) in reshaping genome-wide expression patterns under LN conditions, revealing multi-level regulatory networks [10].

Long non-coding RNAs (lncRNAs, >200 nt) are emerging regulators of plant development and stress adaptation [11, 12]. Specifically, these non-coding transcripts are involved in nitrogen stress responses through epigenetic and post-transcriptional regulation. Nitrogen-responsive lncRNAs have been characterized in *Arabidopsis* (35 LN responsiveness lncRNA) [13], *Populus × canescen* (126 LN responsiveness lncRNA) [14], *Hordeum vulgare* (56 LN responsiveness lncRNA) [15], *Zea mays* (894 LN responsiveness lncRNA) [16] with functional studies revealing conserved mechanisms. The most prominently upregulated transcript of *Arabidopsis*, lncRNA T5120, modulates nitrate signaling, assimilation pathways, and vegetative growth [13]. In *Populus × canescen*, differentially expressed lncRNAs (DELs) co-regulate N-metabolic genes encoding nitrate reductase (NR), glutamate dehydrogenase (GDH), and glutamine synthetase (GS) [14] under N deficiency. Cocurrently, in *Hordeum vulgare* (cultivar B968), 40 lncRNAs target 58 microRNAs (miRNAs), suggesting specific miRNA-mediated regulatory pathways [15]. In *Zea mays*, LNC_003272 and LNC_002923 could play pivotal roles in biological processes, as well as potentially participating in inhibiting the effects of miRNAs in response to nitrogen deficiency [16].

The competing endogenous RNA (ceRNA) hypothesis postulates a universal RNA communication network where mRNAs and lncRNAs competitively bind shared miRNAs via conserved miRNA response elements (MREs), enabling mutual expression control [17]. This mechanism establishes developmental and stress-responsive plasticity in plants through ceRNA crosstalk. For example, lnc-NNR4481 negatively regulates miR172c and nodulation via a ceRNA axis and thus this axis has an impact on nitrogen fixation in *Glycine max* [18]. Under LN stress, *Populus canescens* exhibits a coordinated ceRNA network, included 20 DELs, 47 miRNAs, and 143 expressed mRNAs (DEGs), with some of the functional categories of DEGs associated with the regulation of nitrogen metabolism [14].

Under LN conditions (N0.5), we have demonstrated that sugar beet exhibits specific alterations in phenotypic traits (such as root length and root area), physiological indicators (including GS and peroxidase enzyme activities), and protein-coding gene expression profiles compared to normal nitrogen conditions (N5) [19–21]. However, the key regulatory responses, especially those mediated by lncRNAs as pivotal regulators in modulating gene expression at the post-transcriptional level, remain largely uncharacterized. To address this gap, we systematically investigated LN-responsive lncRNAs in sugar beet through comparative transcriptomic analysis of seedlings under normal N (control) and LN conditions. By integrating high-throughput sequencing with ceRNA network construction, we identified DELs, and elucidated their functional linkages to N signaling pathways via ceRNA-mediated post-transcriptional regulation. This study provides insights into lncRNA-driven nutrient stress adaptation and a possible framework for optimizing N use efficiency in sugar beet.

## Materials and methods

### Experimental material and treatment

Sugar beet (*Beta vulgari*s L.) variety ‘780016B/12 superior’ was from the National Sugar Beet Germplasm Mid-term Bank of Heilongjiang University (Harbin, China), and the experiment was carried out in the artificial culture room. Seeds with high germination rate, full grain and uniform size were selected as materials. Growth conditions followed those described by Li et al. [20]. Plants were maintained at 25 ℃ (day)/18 ℃ (night) u nder a 14 h light/10 h dark photoperiod, with 45–55% relative humidity and a light intensity of 200 µmol·m^− 2^·s^− 1^. When the second pair of true leaves emerged (14 days post-germination), seedlings were moved to modified Hoagland nutrient solution under low nitrogen (LN: N0.5, 0.5 mmol/L) or control (CV: N5, 5 mmol/L) conditions. Foliage (A) and roots (B) were collected after 12 h of LN treatment for early response detection [20], then frozen in liquid N and stored at − 80 °C, respectively.

### Transcriptome sequencing

Total RNA was extracted from sugar beet roots and foliage using RNA-easy Isolation Reagent (Vazyme R701, Vazyme, Nanjing, China) with six pooled seedlings per biological replicate. RNA concentration, OD_260_/OD_230_ and OD_260_/OD_280_ were determined. RNA-specific library construction and sequencing were performed. For mRNA sequencing, poly(A) + RNA enrichment and fragmentation-based library construction were performed according to Li et al. [20], followed by 150-nt paired-end Illumina sequencing. LncRNA sequencing utilized total RNA with rRNA depletion on the same platform. MiRNA libraries were prepared by size-fractionating small RNAs (18–30 nt), ligating 3’/5’ adapters, reverse-transcribing into cDNA libraries, and sequencing on Illumina platforms [22]. A total of 12 samples representing treatments (CV-A1, CV-A2, CV-A3, CV-B1, CV-B2, CV-B3, LN-A1, LN-A2, LN-A3, LN-B1, LN-B2, LN-B3) were sequenced. Transcripts were mapped to the sugar beet reference genome (RefBeet v1.2.2), enabling data analysis to identify lncRNAs, as well as mRNAs [20] and miRNAs [22].

### LncRNA identification and differential expression analysis

Transcripts assemblies from all samples were merged using Cufflinks [23], followed by removal of transcripts with ambiguous strand orientation. Candidate lncRNAs were identified through: (1) Selection of transcripts with ≥ 2 exons to assess gene structural complexity [24]; (2) Length screening >200 nt; (3) Exclusion of transcripts overlapping annotated exonic regions (Cuffcompare); (4) Coding potential analysis via Coding Potential Calculator (CPC2) [25], Coding-Non-Coding Index (CNCI) [26], and Protein Families Database (PFAM) [27]. Comparative metrics used mRNA data from Li et al. [20] to compare exon counts (reflecting transcriptional architecture), Open Reading Frames (ORFs) length distributions (evaluating coding potential) [28] and Fragments Per Kilobase of exon model per Million mapped fragments (FPKM) expression levels quantified by Cuffdiff [23]. Differential expression analysis between experimental groups was performed via EdgeR [29], implementing stringent thresholds (q-value < 0.05) to identify significant differentially expressed lncRNAs (DELs).

### Target prediction and functional enrichment of LncRNAs

Biological functions of DELs were inferred through cis/trans regulatory analysis. Protein-coding genes within 100 kb upstream/downstream of DEL loci were identified as potential cis-targets. Co-expressed mRNAs with |Pearson correlation coefficients| >0.95 were considered trans-targets, and negative correlations may indicate inhibitory roles. Functional enrichment analysis was performed on the above associated mRNAs which were differentially expressed (DEGs) under LN [20] using Gene Ontology (GO) (http://www.r-project.org/) and Kyoto Encyclopedia of Genes and Genomes (KEGG) (http://kobas.cbi.pku.edu.cn/) databases. Important functional categories were determined by hypergeometric testing with FDR correction (FDR ≤ 0.05).

### Validation of LncRNA expression by qRT-PCR

Seven DELs were selected for quantitative real-time polymerase chain reaction (qRT-PCR) validation. Reverse-transcription was conducted using lnRcute lncRNA First-Strand cDNA Kit (Tiangen Biochemical Technology Co., Beijing, China). QRT-PCR was performed on a real-time fluorescence quantitative PCR instrument (Thermo Fisher Scientific Instruments, Shanghai, China; QuantStudio™ 1 Plus) using the MagicSYBR Mixture (Jiangsu Canvax Biotech Co., Jiangsu, China). *BvGAPDH* (*BVRB_5g110740*) and *BvU6* (*BVRB_3g062190*) were selected as the references. Primers were listed in Supplementary Table 1. Relative expression levels were calculated using 2^–ΔΔCt^ method [30].

### Construction of CeRNA regulatory network

Putative interactions between differential expressed lncRNAs and miRNAs (DELs-DEMs) were predicted using psRNATarget [31] based on sequence complementarity, allowing fewer than 4 mismatches and no G/U pairs in the lncRNA-miRNA pairing region. Combined with DEMs-DEGs prediction [22], a tripartite ceRNA network was reconstructed in Cytoscape (v3.10.1) by merging miRNA-lncRNA binding affinities and miRNA-mRNA targeting relationships.

Subsequently, a clustering analysis was conducted on the FPKM values of the DEGs within the ceRNA network. Samples and variables were clustered using a hierarchical clustering algorithm with color-mapped data values (red for higher values and blue for lower values). The screening conditions for DEGs were |log_2_(fold change)| ≥ 1, q-value < 0.05 [20].

## Results

### Genome-wide identification and characterization of nitrogen-responsive LncRNAs in sugar beet

**Fig. 1:**
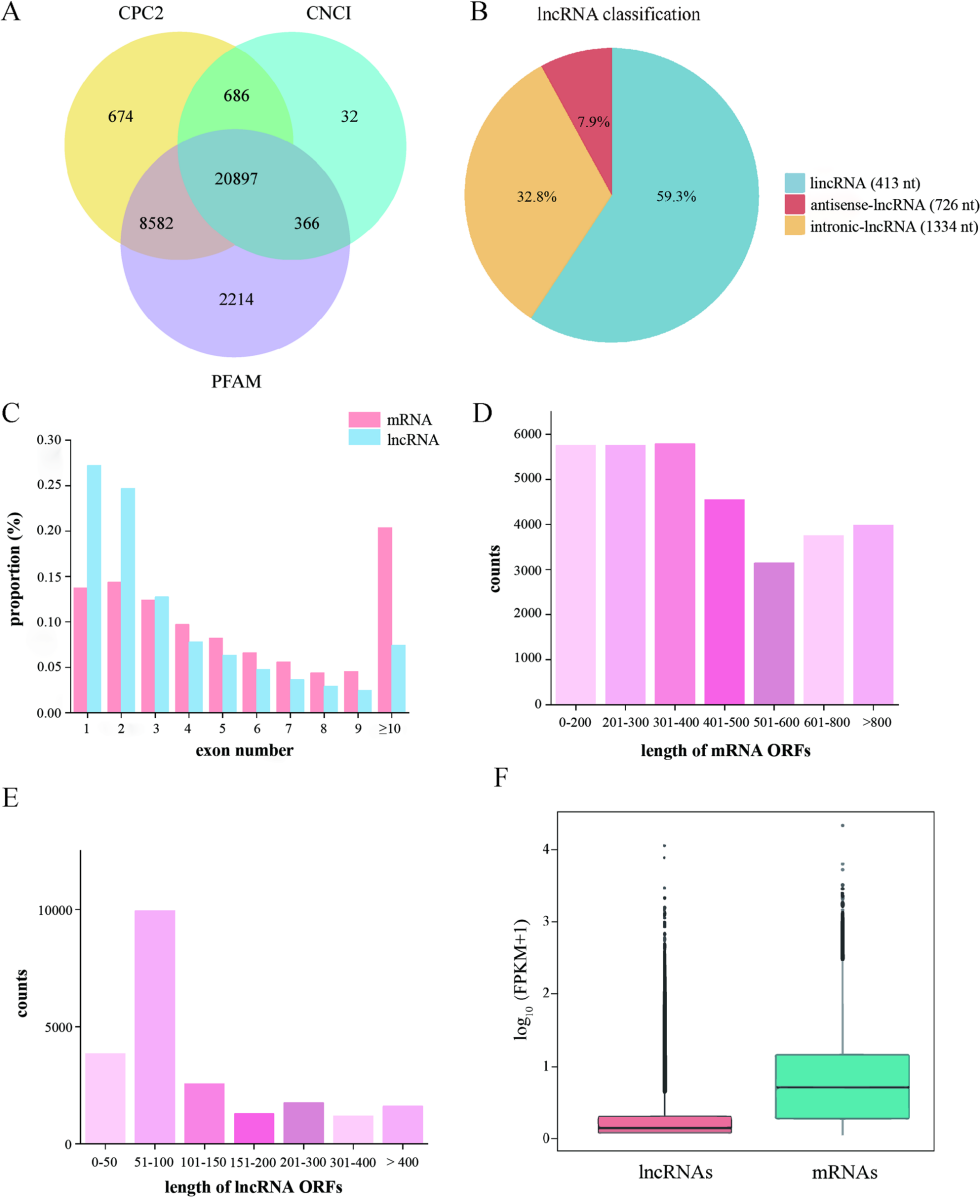
Total lncRNAs in sugar beet including normal N (CV) and low N (LN). **A** Venn diagram showing the numbers of novel lncRNAs identified by Coding Potential Calculator (CPC), Coding Non-Coding Index (CNCI) and Protein Families Database (PFAM). **B** Classification of novel lncRNAs. The average lengths are 413 nt for intergenic lncRNAs (lincRNAs), 1,334 nt for intronic lncRNAs, and 726 nt for antisense lncRNAs. **C** Exon number of lncRNA transcripts and mRNAs. **D** and **E** ORF lengths of mRNAs and lncRNAs, respectively. **F** Comparison of lncRNA and mRNA expression levels [log_10_(FPKM + 1)]

We performed transcriptomic analysis of sugar beet seedlings subjected to normal nitrogen supply (CV, N5: 5 mmol/L N) and low nitrogen treatment (LN, N0.5: 0.5 mmol/L N), with 12 biological replicates representing these treatments. After quality control, yielding 6,134,717 transcripts for lncRNA screening. To reliably identify novel lncRNAs, we employed a combined screening approach using three computational tools (CPC2, CNCI, and PFAM). Transcripts consistently supported by all three methods were considered high-confidence candidates, yielding a final set of 20,897 unknown lncRNAs (Fig. 1A). Those identified by only one or two tools were excluded to ensure stringency. Additionally, 1,341 transcripts with significant homology to known lncRNAs were also excluded from the novel lncRNA set to focus our subsequent analysis on previously uncharacterized molecules. Among these novel lncRNAs, genomic localization revealed intergenic lncRNAs (lincRNAs) as the predominant class (59.3%, *n* = 12,385), followed by intronic lncRNAs (32.8%, *n* = 6,852) and antisense lncRNAs (7.9%, *n* = 1,660) (Fig. 1B); lincRNAs had the shortest average length of 413 nt and intronic lncRNAs had the longest average length of 1,334 nt.

Comparative genomic analysis revealed structural divergence between lncRNAs and coding transcripts. While 52% of lncRNA transcripts contained ≤ 2 exons (vs. 28.1% of mRNAs), only 7.5% spanned > 10 exons compared to 20.4% of mRNAs (Fig. 1C). ORF length distributions further distinguished these RNA classes: lncRNAs peaked at 51–100 nt, contrasting with mRNA ORFs predominantly spanning 101–400 nt (Fig. 1D-E). Expression quantification confirmed FPKM value of lncRNA is lower than that of mRNA (Fig. 1F), aligning with their non-coding, yet regulatory roles. We randomly screened 100 lncRNAs from sugar beet for blast comparison with *Spinacia oleracea* and *Arabidopsis thaliana*. The majority exhibited over 60% homology with *Spinacia oleracea* lncRNAs and more than 50% with those of *Arabidopsis thaliana*, indicating a high degree of conservation in sugar beet (data not shown).

### Identification of LN-responsive DELs

**Fig. 2:**
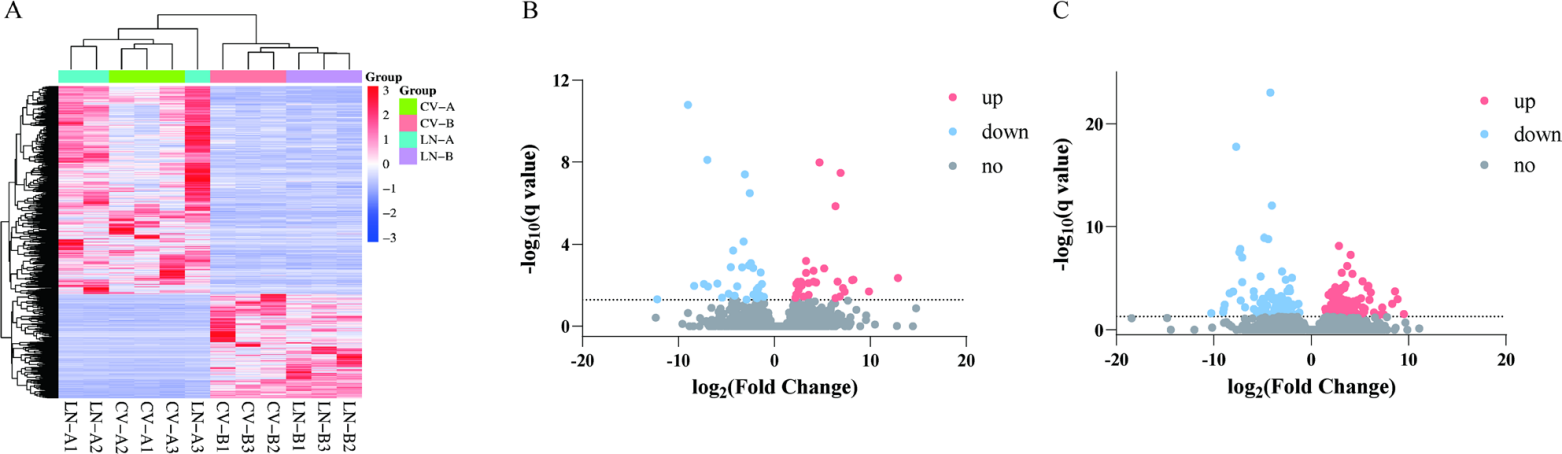
LN-responsive differentially expressed lncRNAs (DELs). **A** Hierarchical clustering of DELs under LN and CV conditions. CV-A, control group, foliage; CV-B, control group, roots; LN-A, LN, foliage; LN-B, LN, roots. Three replicates were performed. **B** and **C** Volcano plots of DELs in foliage and roots of sugar beet. Significantly regulated transcripts (q < 0.05) are highlighted (red: upregulated; blue: downregulated)

Comparative analysis of foliage and root lncRNA expression between LN and normal N supply identified LN-responsive transcripts using stringent thresholds (q-value < 0.05). Hierarchical clustering revealed distinct lncRNA expression patterns between treatments (Fig. 2A, Supplementary Table 2). A total of 120 expressed lncRNAs (DELs) were obtained in foliage, with 66 upregulated and 54 downregulated (Fig. 2B), while 254 DELs were identified in roots, including 116 upregulated and 138 downregulated (Fig. 2C). The comparative analysis revealed that sugar beet roots exhibited a significantly higher number of DELs than foliage when subjected to N-deficient conditions.

### Validation of DELs expression patterns

qRT-PCR analysis of seven randomly selected DELs demonstrated consistent transcriptional tendency with RNA-seq profiles (Supplementary Fig. 1, Supplementary Table 3), confirming the reliability of transcriptome data for the following functional elucidations.

### Functional annotation of LN-DEL-associated regulatory networks

**Fig. 3:**
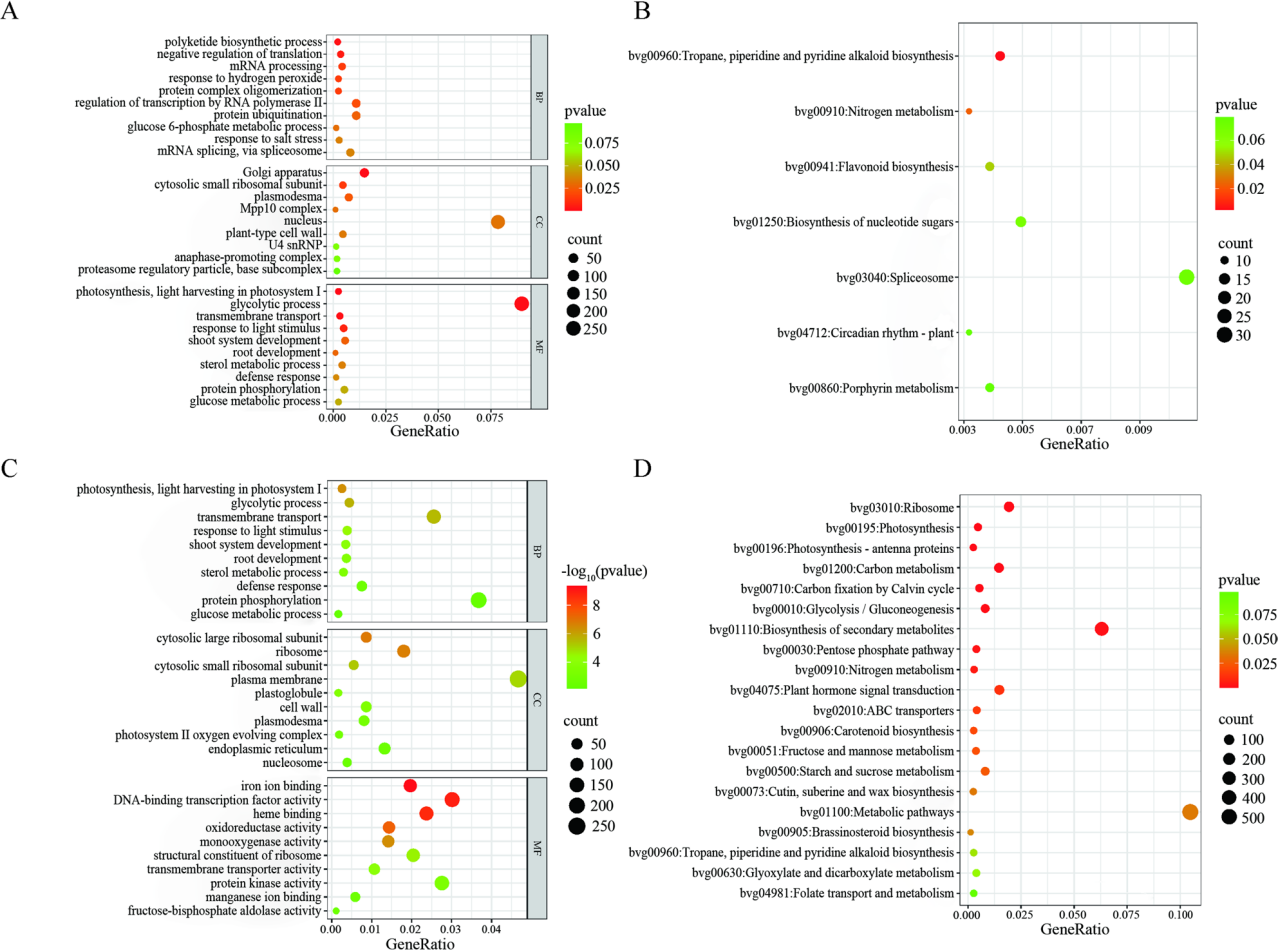
Functional annotation of LN-responsive DEL regulatory networks. **A** Gene Ontology (GO) enrichment of cis-regulatory targets (genomic proximity ≤ 100 kb). **B** KEGG pathway mapping of cis-acting targets. **C** GO profiling of trans-regulatory co-expressed mRNAs. **D** KEGG annotation of trans-acting targets. Significantly enriched terms (FDR < 0.05) are highlighted. The x-axis is the gene ratio; the y-axis is the enrichment of entries. The size of the bubbles represents the number of DELs on the annotated items; the color represents the enrichment degree *p-*value or -log_10_p-value

LncRNAs can correlate with the expression of their neighboring genes, and also act on other genes through base complementary pairing. Cis-target prediction (100 kb flanking DEL loci) identified 2,832 expressed mRNAs (DEGs) associated with 374 LN-DELs (Supplementary Table 4). Trans-target prediction based on the expression correlation between lncRNAs and mRNAs, with a |Pearson correlation coefficient| >0.95, identified 5,441 DEGs (Supplementary Table 5). Among the trans-targeted genes, 71.55% were negatively regulated, compared to 33.5% of the cis-targeted genes, which were positively regulated by lncRNAs. Functional annotation of these targets indicated critical biological processes and metabolic pathways (Fig. 3). GO enrichment highlighted five dominant functional clusters: protein binding (GO: 0005515), plasma membrane (GO: 0005886), nucleus (GO: 0005634), Golgi apparatus (GO: 0005794) and regulation of transcription by RNA polymerase II (GO: 0006357). KEGG pathway analysis further demonstrated metabolic landscape. Among these, we focus on four pathways: nitrogen metabolism (bvg00910), which is directly related to nitrogen adaptation mechanisms and is enriched in the ammonium assimilation core enzyme glutamine synthetase leaf isozyme, chloroplastic (*GS2*,* BVRB_001510*, downregulated); plant hormone signal transduction (bvg04075), which plays a crucial role in nutrient stress; carbon metabolism (bvg01200); and biosynthesis of secondary metabolites (bvg01110), which are closely linked to carbon and nitrogen balance. This systematic annotation establishes a multi-layered regulatory architecture where LN-responsive lncRNAs correlate with both structural (membrane/nucleus) and metabolic (C/N assimilation) adaptations through distinct cis/trans regulatory modes. It is also closely linked to hormone levels.

### Plant hormone signal transduction reprogramming mediated by LN-responsive DELs

**Fig. 4:**
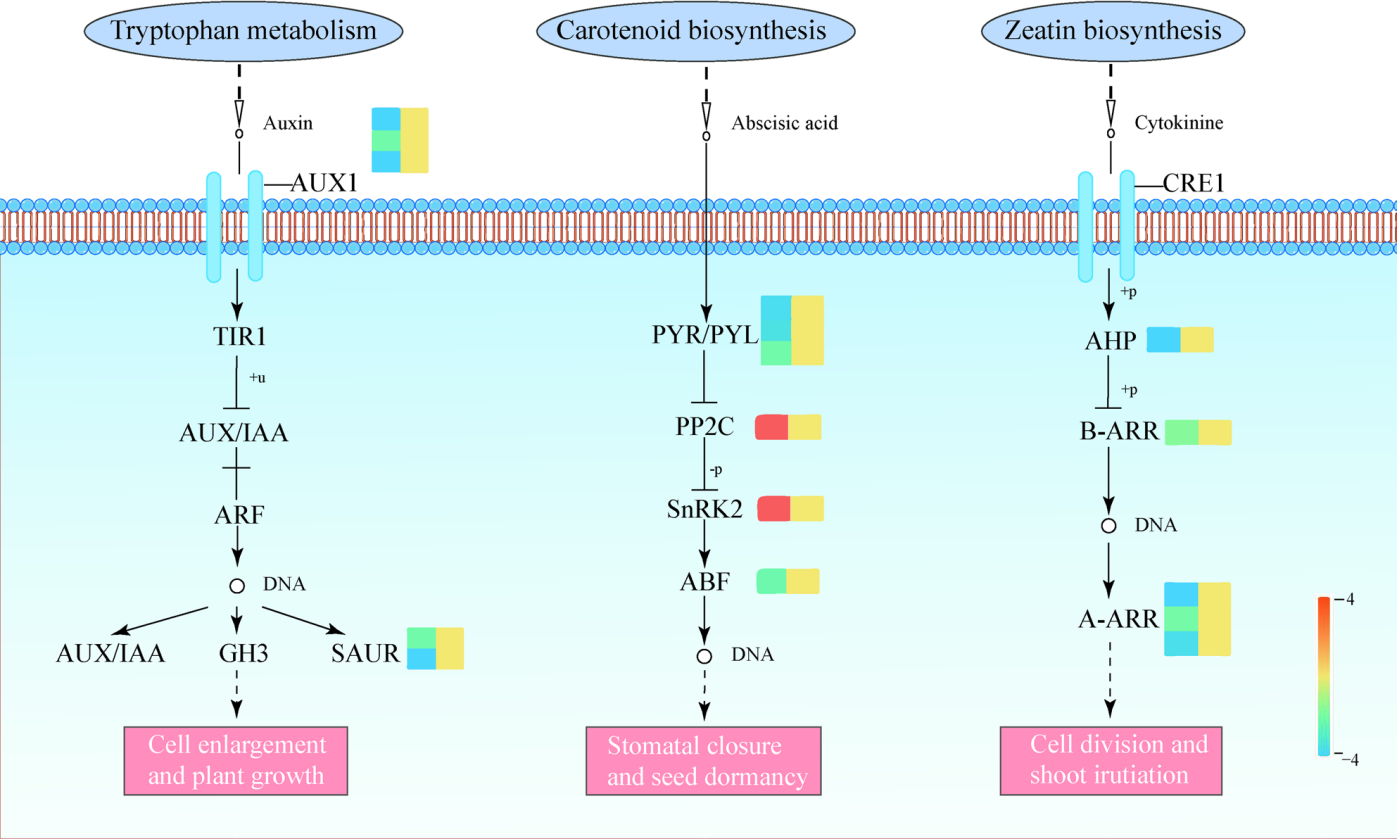
Trans-regulatory DEGs of LN-responsive lncRNAs in hormone signal transduction pathways. Schematic of three plant hormone signaling pathways: Auxin (from tryptophan metabolism) regulates cell enlargement/growth via the TIR1-AUX/IAA-ARF pathway; Abscisic acid (ABA, from carotenoid biosynthesis) mediates stomatal closure/seed dormancy through the PYR/PYL-PP2C-SnRK2-ABF cascade; Cytokinin (from zeatin biosynthesis) promotes cell division/shoot initiation via the CRE1-AHP-B-ARR-A-ARR module. Color intensity of heatmap corresponds to |log_2_FC| of the trans-targets (red: upregulation; blue: downregulation)

Phytohormones serve as central integrators of nutrient stress responses, coordinating growth-defense trade-offs under nitrogen deficiency. Given the significant enrichment of hormone signaling components among trans-targets of LN-responsive lncRNAs (KEGG pathway bvg04075, FDR < 0.05), we annotated this specific pathway (Fig. 4, Supplementary Table 6) to explore their relationship under LN supply condition by analyzing the interaction between these regulators in the coordination of hormones to adapt to stress changes.

LN-responsive DEGs reveal coordinated dysregulation of key hormonal signaling. Our identification of 66 LN-responsive DEGs points to significant alterations in core hormonal pathways under nitrogen deficiency. Crucially, downregulation dominated multiple pathways: auxin transport (auxin transporter-like proteins [AUX; *BVRB_2g024010*, *BVRB_1g000210*, *BVRB_5g111820*]) and signaling (Small auxin-up RNA, SAUR), cytokinin signaling (*BVRB_9g225090* and other 4 genes), and specific components of ABA signaling (abscisic acid receptor *PYL4*). This widespread suppression, alongside mixed regulation in ABA (2 up-regulated, 4 down-regulated including *PYL4*,* BVRB_2g044620*) and gibberellin (1 up-regulated, 1 down-regulated) pathways, suggests a complex LN-induced reprogramming of hormone network. Significantly, several affected pathways (auxin, cytokinin, gibberellin) involve ubiquitin-mediated degradation. This pattern implicates LN-responsive lncRNAs in post-transcriptional modulation of hormone-related genes, potentially through coordinated protein degradation and hormone cross-talk, to mediate sugar beet growth adaptation to LN stress.

### LncRNA-mediated CeRNA regulatory networks in sugar beet under LN

**Fig. 5:**
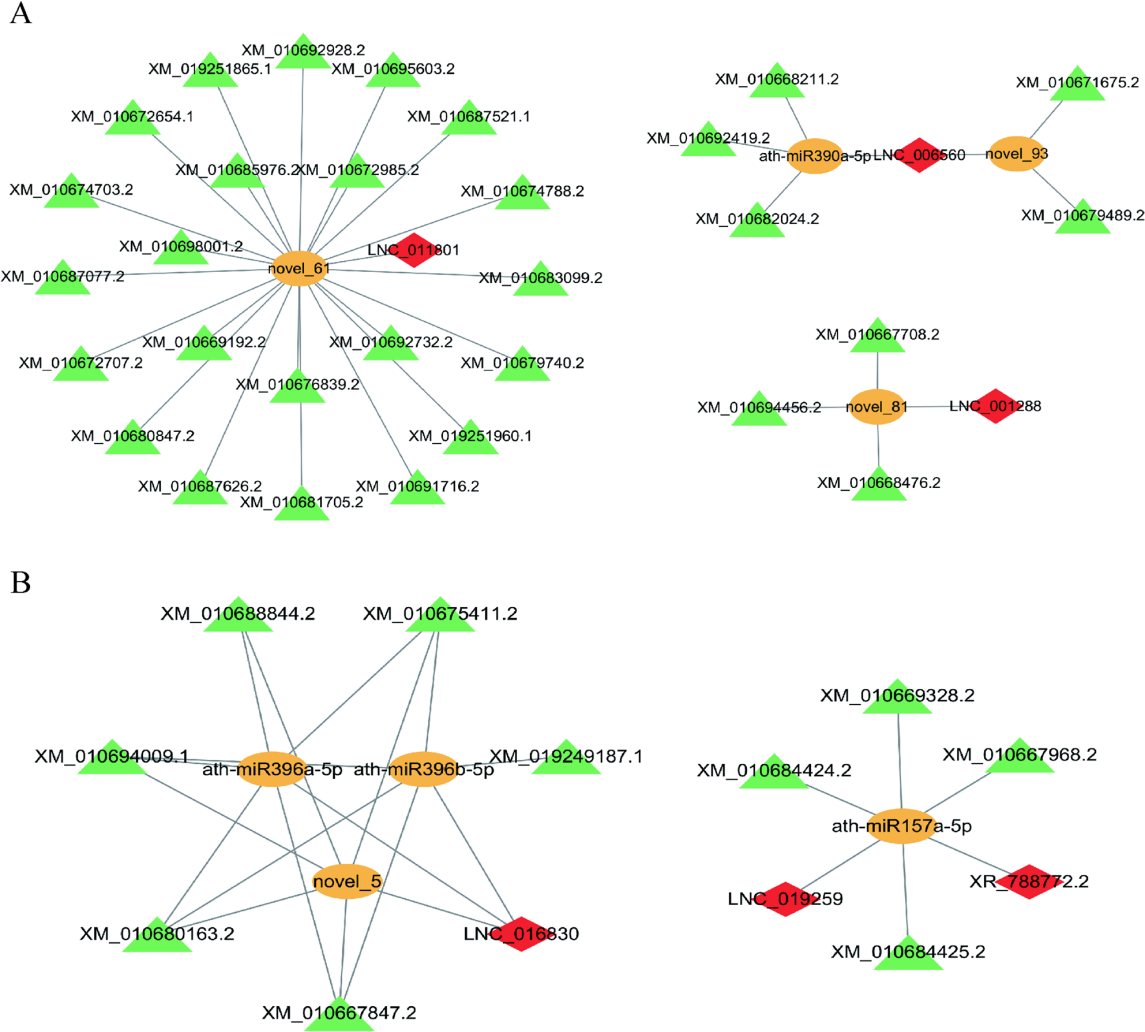
ceRNA regulatory networks in sugar beet under LN. **A** and **B** lncRNA-miRNA-mRNA interaction network in foliage and roots, respectively. The red diamonds represent DELs; the green triangles are DEGs; and the yellow circles indicate DEMs

Phytohormonal and metabolic reprogramming under low nitrogen requires precise spatiotemporal coordination between organs. While foliage prioritizes resource conservation, roots enhance nutrient foraging - a dichotomy potentially orchestrated by competing endogenous RNA (ceRNA) networks. We constructed tissue-specific ceRNA networks by integrating established mRNA and miRNA profiles from our previous transcriptomic dataset responsive to LN [20, 22]. DEL-expressed miRNAs (DEMs) and DEMs-DEGs pairs were predicted according to base sequences and expression levels, respectively. In foliage, the DEL-mediated network comprised 4 miRNA-lncRNA pairs and 30 miRNA-mRNA edges, including 3 lncRNAs, 4 miRNAs, and 30 mRNAs. The high mRNA connectivity (30 edges) indicates extensive transcriptome reprogramming in foliage, while root networks contained 5 miRNA-lncRNAs pairs and 19 miRNA-mRNA connections (3 lncRNAs, 4 miRNAs, and 10 mRNAs) (Fig. 5). It shows that there are fewer but more concentrated interactions in the root, indicating precise regulation through signal hubs that enhances nitrogen foraging. Notably, lncRNA LNC_016830 is a root-enriched lncRNA, emerged as a hub node targeted by 3 miRNAs. It dissociates the auxin-sensitive factor *GRF7/9* (*BVRB_000250*,* BVRB_1g019700*) by binding to miRNA, thus affecting root growth. Meanwhile, miRNA novel_61 demonstrated the highest targeting ability by binding lncRNA LNC_011801, showing broad regulatory potential through targeting the most DEMs to redirect the carbon source under LN stress.

**Fig. 6:**
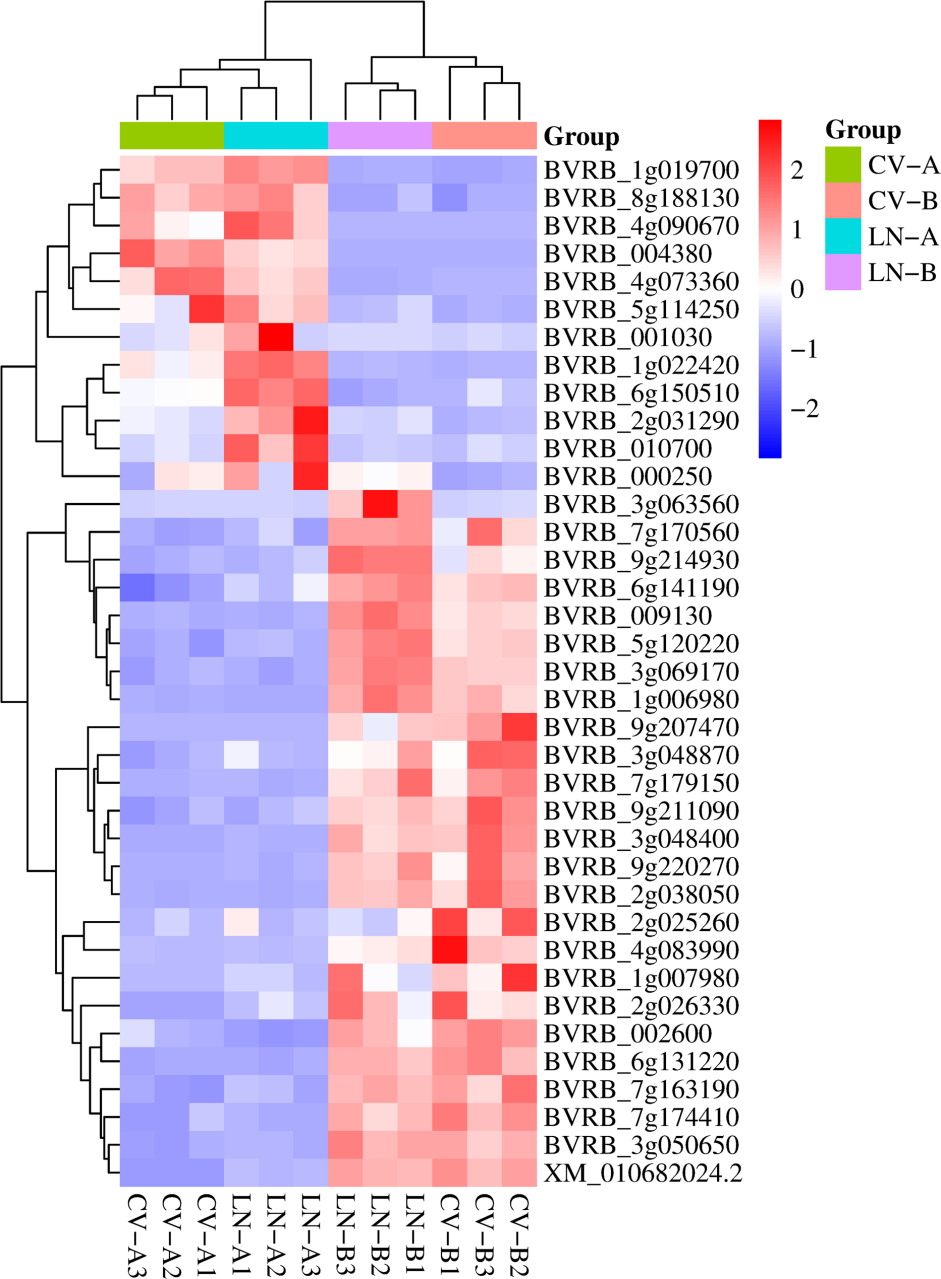
Expression profiles of ceRNA-associated DEGs under LN. Hierarchical clustering of 40 DEGs between LN and control (CV) conditions (|log_2_FC| ≥ 1, q < 0.05). Color gradient reflects transcript abundance (red: up-regulated; blue: down-regulated)

Among 40 ceRNA-associated DEGs, 13 were upregulated and 27 were downregulated under LN including *GRF7*, *GRF9*, *CRWN3* (*BVRB_6g141190*), *RAN1* (*BVRB_7g163190*), *GCN5* (*BVRB_5g120220*), *SR160* (*XM_010682024.2*) (FDR < 0.05) (Fig. 6, Supplementary Table 7). Functional annotation revealed most of these DEGs were related to phytohormones, suggesting lncRNAs coordinate hormonal homeostasis through ceRNA-mediated post-transcriptional control.

## Discussion

### LncRNAs extensively participate in the response of sugar beet to nitrogen deficiency

Emerging evidence highlights lncRNAs as molecular architects of plant stress adaptation [32, 33], yet their role in nitrogen (N) sensing and signaling has remained unclear in non-model crops. While conserved mechanisms like DANA1-mediated drought tolerance [34] and FLAIL-dependent flowering control [35] illustrate lncRNA function in *Arabidopsis.* Our study establishes a systematic framework for lncRNA-mediated N adaptation in sugar beet: a crop species which possesses a specialized N demand strategy [36].

Consistent with lncRNAs in *Arabidopsis* and salt-stressed sugar beet [37], the identified transcripts exhibit structural minimalism (fewer exons and no coding ability, shorter ORFs) and low expression (Fig. 1C-F), suggesting evolutionary selection for rapid stress-responsive regulation. The predominance of lincRNAs (59.3%) aligns with reports in *Populus tomentosa* [38] and *Hordeum vulgare* [15], indicating conserved genomic organization of intergenic lncRNAs across plants.

The identification of 22,238 lncRNAs (Fig. 1A and B) exhibiting reduced lncRNA transcripts exon complexity, shorter ORF lengths and lower expression levels (Fig. 1C-F), similar to prior observations in salt-stressed sugar beet [37]. These features collectively suggest an evolutionary selection for structural minimalism to enable rapid LN responsive regulation in sugar beet. Critically, our analysis revealed a pronounced root-preferential lncRNA reprogramming among the DELs during the early phase of LN response (12 h post-treatment; Fig. 2B and C). Given roots are the principal site of nitrogen perception and uptake, this spatial specificity highlights the central role of root lncRNAs in orchestrating the initial nitrogen sensing-signaling crosstalk essential for rapid adaptation to nutrient stress.

### Metabolic coordination and hormonal crosstalk in sugar beet under low nitrogen

Comparative analysis revealed trans-regulation as the dominant lncRNA regulatory paradigm in the nitrogen stress response of sugar beet (trans/cis target ratio = 3.47:1), with 1,129 trans-targets converging on carbon (bvg01200) and nitrogen (bvg00910) metabolic pathways (Fig. 3, Supplementary Tables 4–5). It indicates that lncRNA-mediated target genes are involved in C/N metabolic homeostasis under low nitrogen conditions. This lncRNA-mediated C/N coupling mechanism has also been observed in *Populus tomentosa* [38] and *Oryza sativa* [39], suggesting that this mechanism is a widely conserved strategy for plants to respond to low nitrogen stress, coordinate resource allocation, and maintain survival and growth [40]. Later, we identified a key gene, chloroplast glutamine synthetase *GS2*, which is a hub node targeted by XR_791134.2 and LNC_011801 in the nitrogen metabolism pathway. As the important enzyme catalyzing ammonium to glutamine [41, 42], *GS2*’s lncRNA-mediated regulation may fine-tune ammonium detoxification efficiency under LN stress, mirroring post-translational GS modulation mechanisms reported in Fabaceae species [43].

In response to LN condition, phytohormonal reprogramming represents a crucial adaptation mechanism in sugar beet. This reprogramming involves the coordination of auxin (IAA), cytokinin (CTK), and abscisic acid (ABA) signaling cascades by lncRNAs (Fig. 4), aligning with previous reports on N stress responses [44, 45]. Specifically, as far as growth hormone is concerned, the lncRNA-mediated coordination of auxin signaling includes the suppression of auxin influx carriers (*AUX2/3/4*) and upregulation of stress-responsive *SAUR* genes under N deprivation (Supplementary Table 6), creating an auxin redistribution signature that prioritizes root plasticity over shoot growth [46]. The induction of *SAUR* genes, a cohort known for promoting cell expansion and modulating auxin transport, is particularly significant. It suggests a mechanistic shift towards enhancing root exploratory capacity, thereby maximizing nitrogen acquisition from a depleted environment. This strategic response is evolutionarily conserved, can be evidenced by the analogous phenomenon in apple rootstocks under low nitrogen stress [47]. The convergence of this mechanism across distantly related species underscores its fundamental role in plant adaptation to nutrient limitation. Ultimately, this lncRNA-directed reprogramming fine-tunes the spatial distribution of auxin, reallocating metabolic resources to reinforce root architecture. Concurrently, CTK signaling components (*AHPs*, *B-ARRs*, *A-ARRs*) were downregulated, indicating potential attenuation of CTK signaling. Notably, ABA signaling exhibited distinct regulatory patterns in sugar beet under LN stress conditions. ABA receptor *PYL4* was upregulated, while *PP2C* was downregulated, activating the ABA signaling cascade, where PYL proteins inhibit *PP2C* activity to enable downstream signal transduction [48, 49]. This tripartite hormonal regulation-IAA-mediated architectural remodeling, CTK-dependent growth modulation, and ABA-driven stress priming-collectively governs the metabolic balance of sugar beet under LN conditions through lncRNA-mediated transcriptional control.

### The CeRNA network integrates hormonal and nitrogen signaling

LncRNAs exhibit diverse modes of action with miRNAs, acting both as miRNA precursors and as miRNA regulators [50], regulating stress tolerance at the posttranscriptional level. The competing endogenous RNA (ceRNA) hypothesis suggests that different RNA classes (i.e., lncRNAs and mRNAs) compete for shared miRNAs, to add further complexity to gene expression regulation [15]. The sugar beet ceRNA landscape under LN stress reveals a multi-tiered regulatory architecture, with foliage (4 miRNAs/4 lncRNAs/30 mRNAs) and root (3 lncRNAs/4 miRNAs/10 mRNAs) networks converging on hormone signal transduction (Fig. 5, Supplementary Table 7). Specifically, LNC_016830, targeted by miR396a/b-5p, regulates transcription factors *GRF7* (*BVRB_000250*) and *GRF9* (*BVRB_1g019700*), which modulate auxin sensitivity [51]. Crucially, key target mRNAs within this ceRNA network represent multiple phytohormone pathways and demonstrate the integration of hormonal and nitrogen signaling mediated by lncRNAs. These include: *GRF7* (regulated by ABA signaling [52]); *CRWN3* (*BVRB_6g141190*), which is involved in *ABI5* degradation within the ABA pathway [53]; *RAN1* (*BVRB_7g163190*), which participates in ethylene signaling through copper delivery [54]; and *GCN5* (*BVRB_5g120220*), which is associated with gibberellin biosynthesis [55], collectively highlight how ceRNA-mediated regulation integrates hormonal signaling (ABA, ethylene, gibberellin) with nitrogen responses via lncRNAs (Fig. 6).

*SR160* (*XM_010682024.2*), a homolog of brassinosteroid-insensitive 1 (BRI1), functions as a brassinosteroid receptor influencing plant growth [56]. This indicates that *SR160* may act as a central regulator coordinating LN adaptation through brassinosteroid-responsive lncRNAs. Among the 40 ceRNA-associated mRNAs, 27 were downregulated in sugar beet under LN conditions, with a predominance of hormone-related genes, supporting the notion that nitrogen deficiency inhibits growth via hormonal interactions [57, 58]. Collectively, these findings position ceRNAs as molecular regulators that modulate miRNA activity. This modulation potentially amplifies stress signals (ABA/ethylene) while concurrently dampening plant growth pathways (auxin/gibberellin/brassinosteroid), thereby orchestrating metabolic and developmental adaptations to nitrogen deficiency.

## Conclusions

This study identifies low nitrogen (LN)-responsive lncRNAs in sugar beet, with 120 and 254 differentially expressed lncRNAs (DELs) in foliage and roots, respectively. These DELs coordinate nitrogen metabolism, transmembrane transport, and plant hormone signal transduction through 1,454 lncRNA-mRNA pairs (trans/cis = 3.47:1). Specifically, the pivotal regulators, lncRNA XR_791134.2 and LNC_011801 may be critically involved in *GS2*-mediated ammonium assimilation and auxin transporter suppression. Tissue-specific ceRNA networks serve as a central platform to integrate nitrogen sensing with hormonal reprogramming, exemplified by LNC_016830 which interacts with miR396a/b-5p to regulate *GRF7/9* and coordinates ABA signaling via *CRWN3*-mediated *ABI5* degradation. These findings establish lncRNAs as master regulators of nitrogen-dependent developmental plasticity, providing molecular targets for breeding high nitrogen use efficiency sugar beet cultivars.

## Supplementary Information

Supplementary Material 1.

Supplementary Material 2.

## Data Availability

The transcriptome and genomic data during the current study are available in the NCBI repository. The names of the repository/repositories and accession number(s) can be found below: NCBI SRA database, accession number SRP422868.
