# Supplementary material for: LncRNA-mediated early regulatory networks in sugar beet (Beta vulgaris L.) response to low nitrogen
Source: BMC Genomics. 2025 Nov 14;26:1112. doi: 10.1186/s12864-025-12301-6 (PMC12717752; doi:10.1186/s12864-025-12301-6)
Supplement: Supplementary file 1 — Supplementary Material 1. [file 12864_2025_12301_MOESM1_ESM.docx]

# Supplementary Figure

# Supplementary Figure 1

**
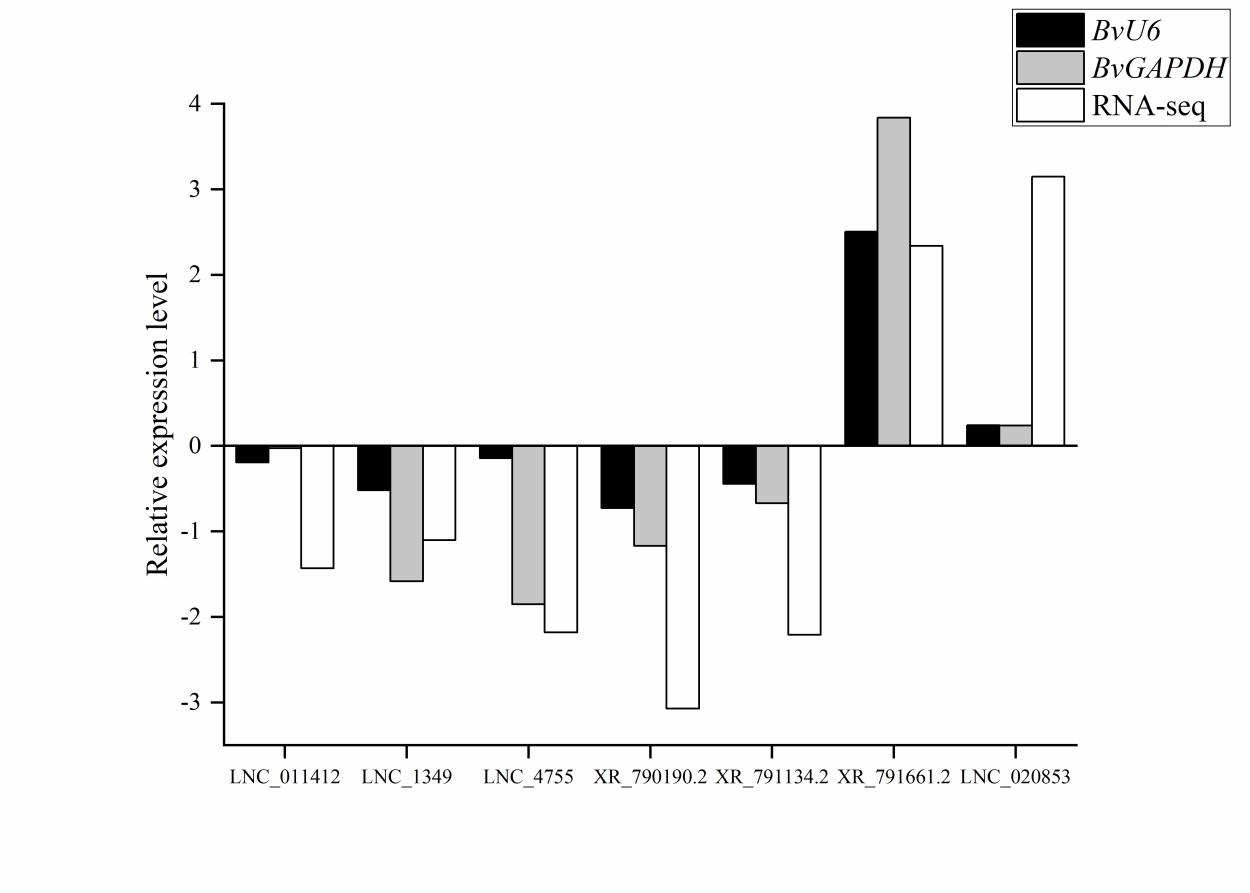
**

**Supplementary Figure 1** Validation of DELs expression consistency between qRT-PCR and RNA-seq in sugar beet under LN conditions. Black bars: *BvU6* internal reference. Grey bars: *BvGAPDH* internal reference.
